# MicroRNA-490-3p inhibits migration and chemoresistance of colorectal cancer cells via targeting TNKS2

**DOI:** 10.1186/s12957-021-02226-1

**Published:** 2021-04-13

**Authors:** Jing Li, Rubing Mo, Linmei Zheng

**Affiliations:** 1 Department of Emergency Surgery, Hainan General Hospital, Hainan Affiliated Hospital of Hainan Medical University, Haikou, 570311 Hainan Province China; 2 Department of Pneumology, Hainan General Hospital, Hainan Affiliated Hospital of Hainan Medical University, Haikou, 570311 Hainan Province China; 3 Department of Obstetrics, Hainan General Hospital, Hainan Affiliated Hospital of Hainan Medical University, Haikou, 570311 Hainan Province China

**Keywords:** Colorectal cancer, TNKS2, miR-490-3p, Chemoresistance, Migration

## Abstract

**Objective:**

Colorectal cancer is one of the most common malignancy in the world. The oncogenesis of colorectal cancer is still not fully elucidated. It was reported that microRNA-490-3p (miR-490-3p) was closely related to the regulation of cancers. However, if miR-490-3p could also affect colorectal cancer and the specific mechanism remains unclear.

**Methods:**

qRT-PCR was conducted to examine the expression of miR-490-3p. DIANA, miRDB, and TargetScan databases were used to identify target genes. LOVO and SW480 cells were transfected by miR-490-3p mimics and inhibitors. Transwell assay was used to measure cell invasion and migration. Cisplatin and fluorouracil were administered to investigate chemotherapy resistance. Western blot was used to measure TNKS2 protein expression. Binding sites were verified using the double luciferase assay.

**Results:**

miR-490-3p expression was low in the colorectal cancer cells. The level of miR-490-3p was negatively correlated with cell migration and invasion of cancer cells. miR-490-3p could bind to TNKS2 mRNA 3′UTR directly. miR-490-3p can suppress cell viability and resistance to chemotherapy in colorectal cancer cells through targeting TNKS2.

**Conclusions:**

miR-490-3p could affect colorectal cancer by targeting TNKS2. This study may provide a potential therapeutic target for colorectal cancer.

## Introduction

Colorectal cancer (CRC) is the most pernicious malignancy in the world [1]. The etiology of CRC is complicated, includes genetic and environmental factors [2]. The high recurrence and metastasis rate are the major risks for late-stage CRC patients [3]. In the past decades, efforts have been in developing novel screening techniques and therapeutic agents towards this deadly disease, but a sensitive testing method and more effective therapeutic agents are still needed [4].

MicroRNAs (miRNAs) are non-coding RNAs that contain 22 nucleotides. miRNAs widely participate in essential biological processes in various kinds of cells. miRNAs have been proved to be closely related to the development and progression of tumors. The function of miRNAs in CRC can contribute to its diagnosis and treatment.

microRNA-490-3p (miR-490-3p) was believed to suppress the growth of several types of cancers. It was reported that miR-490-3p could suppress glioblastoma [5], glioma [6], lung [7] and gastric carcinoma [8, 9], breast cancer [10–13], and liver carcinoma [14, 15]. Meanwhile, the epithelial-mesenchymal transition process could be modulated by miR-490-3p [16]. miR-490-3p could stimulate the apoptosis of esophagus cancer through modulating MAPK1 [17]. However, the role of miR-490-3p in CRC remains unclear.

In this study, we investigated the role of miR-490-3p in the cell migration, invasion, and chemoresistance of CRC. Besides, the bioinformatics method was used to identify the potential target of miR-490-3p. This study might reveal the potential underlying mechanisms of CRC.

## Materials and methods

### Clinical samples

One hundred and sixty-two CRC patients were recruited in the research in the Department of Surgery, Hainan General Hospital. During surgery, cancer and paracancerous tissues were collected.

### Bioinformatics methods

miRDB [18], DIANA [19], and TargetScan databases [20] were used to predict the target genes. A Venn diagram was drawn to identify the common genes.

### Cell culture

CRC cell lines (SW480, LOVO, DLD1, SW48, RKO, HCT116, HT29, SW620, HCT8) and normal colon cell line (FHC) were purchased from the Shanghai Huiying Biotechnology. Cells were cultivated with Roswell Park Memorial Institute-1640 medium containing 10% FBS (HyClone, USA) at 37 °C and 5% CO_2_.

### qRT-PCR

TRIzol reagent (Invitrogen) was used to extract total RNA. Reverse transcription was conducted using TaqMan miRNA Reverse Transcript Kit (Applied Biosystems, USA). The PCR was performed on the Prism 7500 FAST Sequence Detection System of Applied Biosystems. Primers used in this study include miR-490-3p (forward: 5′-CGTGGATCCTTCTTCAACCAACGGTGGTG-3′, reverse: 5′-CCAGAATTCAAAGCAGGAAGAGTAAGACTTCC-3′), TNKS2 (forward: 5′- CGCGGATCCTGAAGGTATGGTCGATG-3′, reverse: 5′- CGCGAATTCAATTTAGTACAGACAACCC-3′), PCBP1 (forward: 5′-CAGTGCGGCTCCCTGATTG-3′, reverse: 5′- CCTCTGGAGAGCTGGAGTCAATTC-3′), RASAL2 (forward: 5′-TCCCTCGTGTTCTTGCTGAT-3′, reverse: 5′- GTCTGTGTTGTCCTGGCTTG-3′), and GAPDH (forward: 5′-AACGGATTT GGTCGTATTG-3′, reverse: 5′-GGAA GATGGTGATGGGATT-3′).

### Western blot

Cell proteins were extracted using RIPA lysis buffer and quantified by BCA assay. The proteins were electrophoresed on a polyacrylamide gel and then transferred to polyvinylidene difluoride (PVDF) membranes (Millipore, Billerica, MA, USA). The membranes were coated with a primary antibody (Abcam, UK) overnight at 4 °C. After rinsing with the tris-buffered saline Tween, proteins were cultured with the secondary antibody (Abcam, UK) at room temperature for 2 h. Then, an ECL chemiluminescent kit (Advansta, USA) was used to expose protein bands.

### Cell proliferation

Cells (1×10^4^ cells/well) were plated into 96-well plates and cultured by different treatments. Cell Counting Kit-8 (CCK-8; Nanjing Jiancheng, Nanjing, China) reagent (10 μL) was added to each well. Then, cells were incubated at 37°C for 2 h, and absorbance was recorded at 450 nm by a microplate reader.

### Transwell assays

Transwell assay was used to examine the ability of cell migration and invasion. The upper and lower chambers were separated by placing 8-μm pore size inserts. 1×10^5^ cells were added into the upper chambers (Corning, Lowell, MA, USA). In the bottom chambers, a cell culture medium containing 10% FBS was added. After incubating at 37°C and 5% CO_2_ for 48 h, the cells that migrated into the lower chamber were stabilized in 4% methanol for 20 min, then stained with the 0.1% crystal at room temperature for 10 min. An Olympus microscope was used to count the cells from five randomly selected fields.

### Cell transfection

miR-490-3p mimic (LV-mimics), mimic control (LV-mNC), miR-490-3p inhibitor (LV-inhibitor), and inhibitor control (LV-INC) were purchased from RiboBio (Guangzhou, China). Cells were plated into 8-well plates with a density of 2×10^5^ cells per well. Lipofectamine 2000 (Invitrogen, USA) was used to package the miR-490-3p mimics and miR-490-3p inhibitor plasmids into cells according to the instructions of the manufacturer.

### Dual-luciferase reporter assay

Cells were digested using trypsin and seeded into 24-well plates before transfection. miR-490-3p-WT plasmid and miR-490-3p-MUT plasmid or TNKS2-WT/MUT plasmid were mixed with culture medium. A transfection reagent was added, and the mixture was incubated for 48 h. Signals were identified by utilizing a Dual-Luciferase Reporter Assay Kit (Promega, USA) following the protocols. Transfection efficiency was observed by a fluorescence microscope (Leica, Wetzlar, Germany).

### Statistical analysis

Experimental data were expressed as mean ± standard deviation. Statistical analyses were performed using SPSS 25 (Chicago, IL, USA). The Student *t*-test was used to analyze the correlation between the two groups. ANOVA was used to analyze among more than two groups. Spearman’s rank test was utilized for analyzing the correlation of miR-490-3p and TNKS2. Experiments were conducted at least three times. *p*<0.05 was considered statistically significant.

## Results

### Significant low expression of miR-490-3p in the CRC tissues

**Fig. 1:**
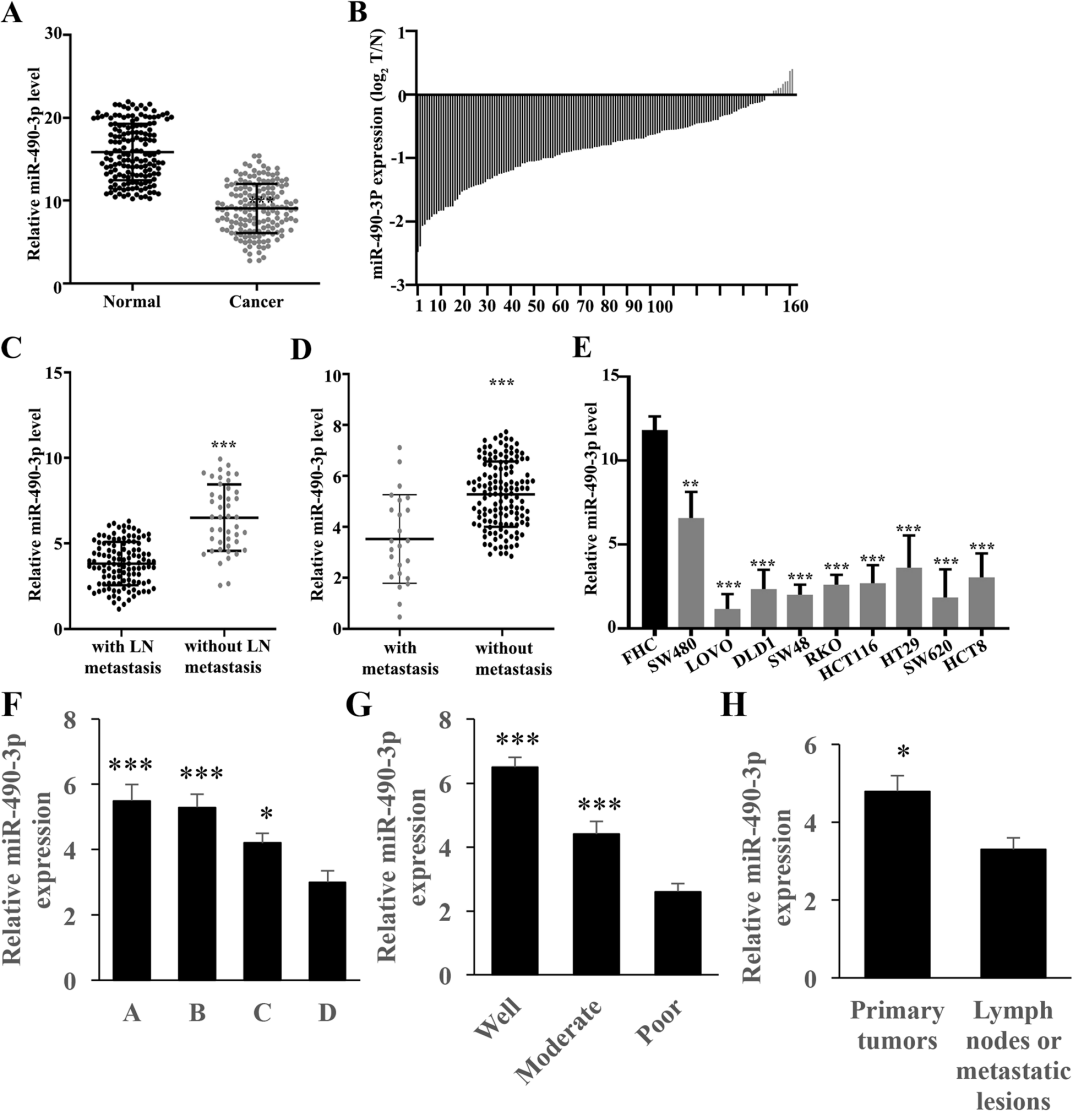
Significant low expression of miR-490-3p expression in colorectal cancer tissues and cell lines. **a** The expression of miR-490-3p was significantly decreased in 162 colorectal cancer samples (*p*<0.001). **b** Low expression in 150 tumor tissues and high expression in 12 tumor tissues. **c** miR-490-3p expression was lower in tissues with lymph node metastasis (*p*<0.001). **d** miR-490-3p expression in tissues with distant metastasis was lower than that without distant metastasis (*p*<0.001). **e** Significant low expression of miR-490-3p expression in colorectal cancer cell lines (*p*<0.001); **f** miR-490-3p expression was significantly higher in tissues of Dukes stages compared with Dukes (*p*<0.001); **g** miR-490-3p expression was significantly higher in the well or moderately differentiated tissues compared with poorly differentiated tissues (*p*<0.001); **h** miR-490-3p expression was significantly higher in the primary tumor tissues compared with lymph nodes or metastatic lesions (*p*<0.05)

A significantly lower level of miR-490-3p in 162 CRC samples (*p*<0.001) was observed compared to normal tissues (Fig. 1a). Figure 1 b revealed low expression in 150 tumor tissues and high expression in 12 cancerous tissues. We examined the expression of miR-490-3p in CRC patients with lymph node metastasis and patients without lymph node metastasis (Fig. 1c) and found that the expression of miR-490-3p in CRC patients with lymph node metastasis was remarkably lower compared to the patients without lymph node metastasis (*p*<0.001) (Fig. 1c). The level of miR-490-3p in CRC specimens with and without distant metastasis was also measured. The level of miR-490-3p in tissues with distant metastasis was markedly lower than that without distant metastasis (*p*<0.001) (Fig. 1d). Meanwhile, the expression of miR-490-3p in several types of CRC cell lines and normal colon cell lines was also measured. A significantly lower expression of miR-490-3p was observed in all CRC cell lines compared to normal cell lines (*p*<0.001) (Fig. 1e). Besides, the expression of miR-490-3p in the tissues of patients was analyzed based on the Dukes staging. A remarkable higher expression of miR-490-3p in A, B, and C Dukes stages was found compared with the D Dukes stage (Fig. 1f). The levels of miR-490-3p in the well or moderately differentiated tissues were marked higher than that of poorly differentiated tissues (Fig. 1g). Significant lower expression of miR-490-3p in the lymph nodes or metastatic lesions was observed compared with primary tumors (Fig. 1h).

### CRC cell viability was suppressed by miR-490-3p

**Fig. 2:**
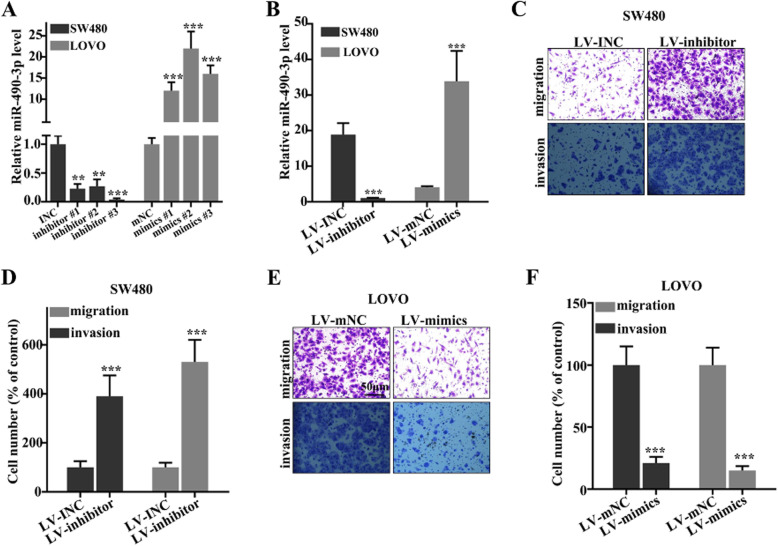
Colorectal cancer cell migration and invasion were suppressed by miR-490-3p. **a** Three mimic sequences and inhibitor sequences were used, and the most effective inhibitor and mimics were selected in the following experiments. **b** The knockdown model of miR-490-3p in SW480 cell line and overexpressed model of miR-490-3p in LOVO cell line were established. **c** The cell migration and invasion of colorectal cancer cells were measured after LV-inhibitor treatment. **d** The cell migration and invasion of colorectal cancer cells were increased significantly after miR-490-3p was knockdown. **e** The cell migration and invasion of colorectal cancer cells were measured after LV-mimics treatment. **f** After treatment with LV-mimics, cell migration and invasion were remarkably suppressed

Knockdown model of miR-490-3p in SW480 cell line and overexpressed model of miR-490-3p in LOVO cell line were established (Fig. 2a, b). Transwell assay was used for exploring cell migration and invasion in CRC cells. The cell migration and invasion of CRC cells were increased significantly after miR-490-3p was knockdown (Fig. 2c, d). However, after treatment with LV-mimics, cell migration and invasion were remarkably suppressed (Fig. 2e, f). The suppression of miR-490-3p on the migration and invasion of CRC cells indicated that miR-490-3p might be involved in the inhibition of CRC.

### Chemoresistance of CRC cells was suppressed by miR-490-3p

**Fig. 3:**
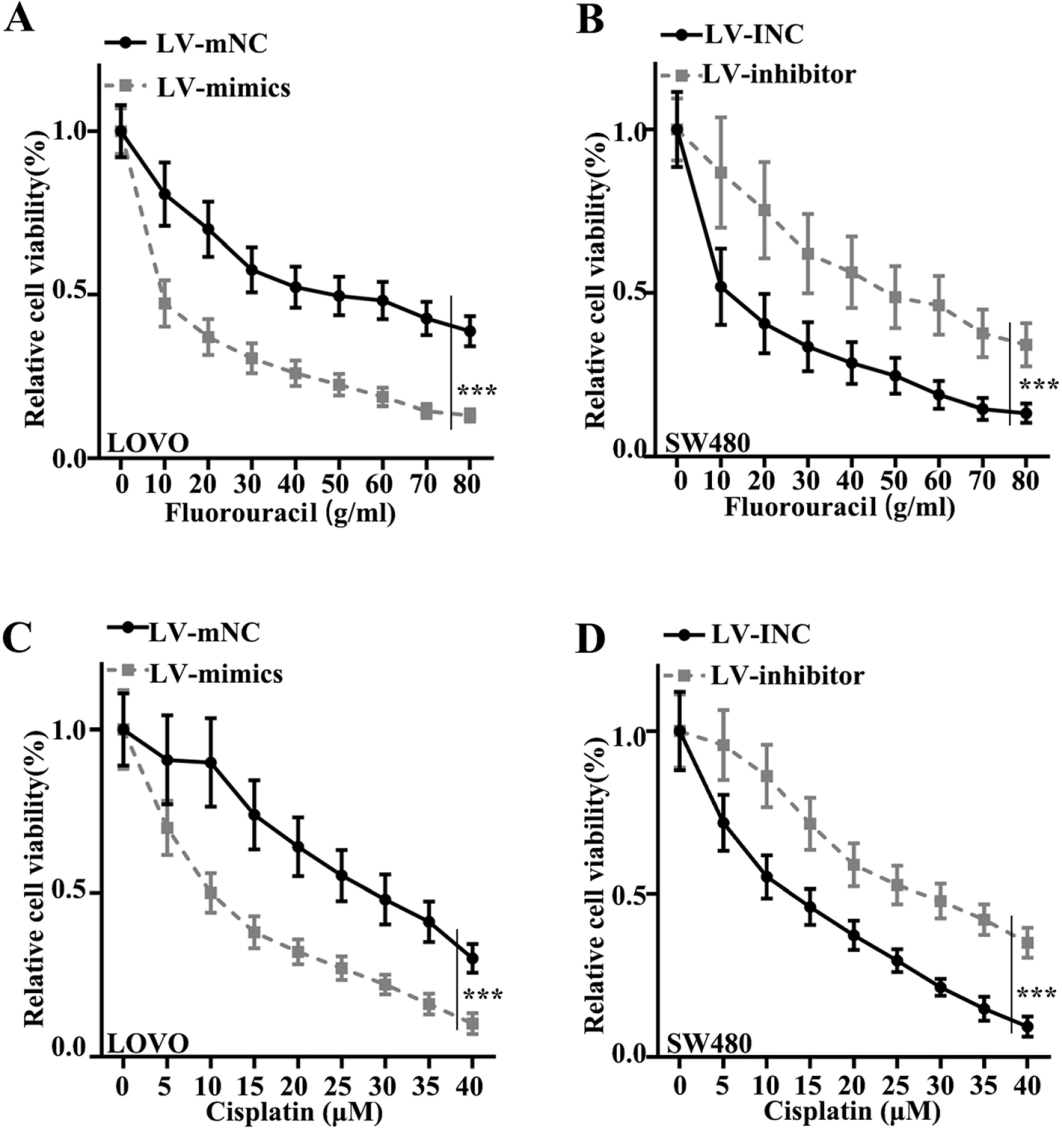
Chemoresistance of colorectal cancer cells was inhibited by miR-490-3p. **a** When treated with fluorouracil, the cell viability of the LV-mimics groups in the LOVO cell lines was lower, in comparison with the LV-mNC group (*p*<0.001). **b** Cell viability of the LV-inhibitor group was higher in SW480 cell lines than that in the LV-INC group (*p*<0.001). **c** When treated with cisplatin, the relative cell viability was higher in the LV-mNC group in LOVO cell lines, compared with that in the LV-mimics group (*p*<0.001). **d** Relative cell viability was downregulated in the LV-INC group in SW480 cell lines, compared with that in the LV-inhibitor group (*p*<0.001)

Fluorouracil and cisplatin were applied to test chemoresistance in CRC cell lines. When treated with fluorouracil, the cell viability of the group LV-mimics in the LOVO cell line was remarkably lower compared with group LV-mNC (*p*<0.001) (Fig. 3a). The cell viability of group LV-inhibitor was significantly elevated in SW480 cell lines than that in group LV-INC (*p*<0.001) (Fig. 3b). When treated with cisplatin, the relative cell viability was markedly higher in group LV-mNC in LOVO cell lines, compared with that in group LV-mimics (*p*<0.001) (Fig. 3c). The relative cell viability was significantly lower in the group LV-INC in SW480 cell lines, compared with that in the group LV-inhibitor (*p*<0.001) (Fig. 3d).

### miR-490-3p was negatively correlated with the expression of TNKS2

**Fig. 4:**
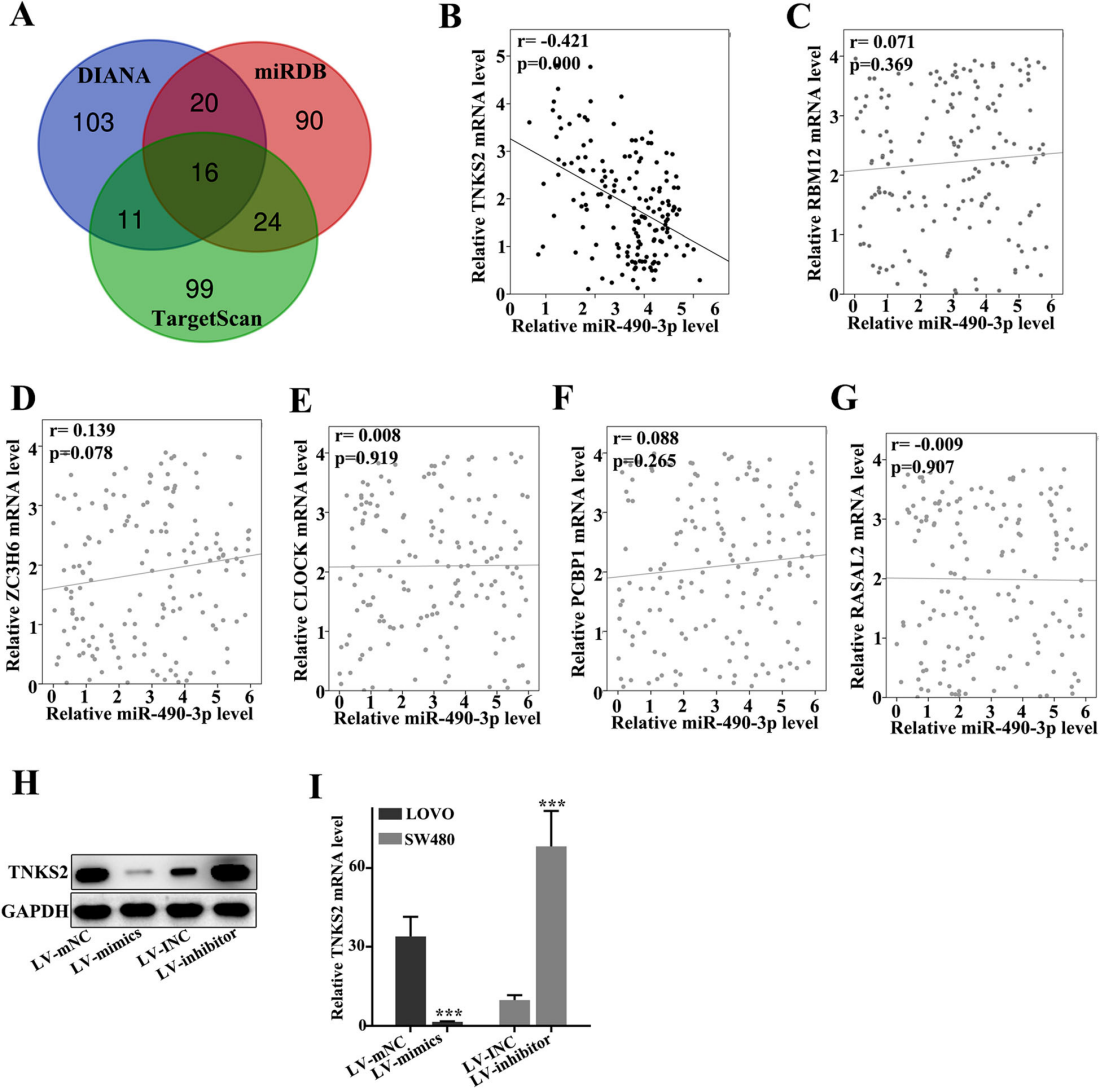
miR-490-3p was negatively correlated with the expression of TNKS2. **a** A total of 16 genes were screened using DIANA, TargetScan, and miRDB databases. **b** The relative mRNA expression of TNKS2 exhibited negative correlation with miR-490-3p (*p*< 0.05). **c** The relative RBM12 mRNA level exhibited no correlation with miR-490-3p (*p*> 0.05). **d** The relative ZC3H6 mRNA level exhibited no correlation with miR-490-3p (*p*> 0.05). **e** The relative CLOCK mRNA level exhibited no correlation with miR-490-3p (*p*> 0.05). **f** The relative PCBP1 mRNA level exhibited no correlation with miR-490-3p (*p*> 0.05). **g** The relative RASAL2 mRNA level exhibited no correlation with miR-490-3p (*p*> 0.05). **h** TNKS2 protein expression was measured. **i** TNKS2 was significantly inhibited in the miR-490-3p overexpressed cell line but increased in the miR-490-3p knockdown cell line (*p*< 0.001)

Three databases (DIANA, TargetScan, and miRDB) were used to identify the target genes of miR-490-3p. A total of 16 genes were screened using the three databases (Fig. 4a). The correlation of these genes with miR-490-3p was measured, and part of the data was presented. The relative mRNA expression of TNKS2 exhibited a negative correlation with miR-490-3p (*p*< 0.05) (Fig. 4b). However, the mRNA levels of RBM12, ZC3H6, CLOCK, PCBP1, RASAL2, and other 10 genes exhibited no correlation with miR-490-3p (Fig. 4c–g). The protein expression of TNKS2 in LOVO and SW480 cell lines was detected after treatment with LV-mimics or LV-inhibitor (Fig. 4h, i). We found that the level of TNKS2 was significantly inhibited in the miR-490-3p overexpress cell line. In contrast, TNKS2 was remarkably increased in the miR-490-3p knockdown cell line (Fig. 4h, i).

### TNKS2 was the downstream target of miR-490-3p

**Fig. 5:**
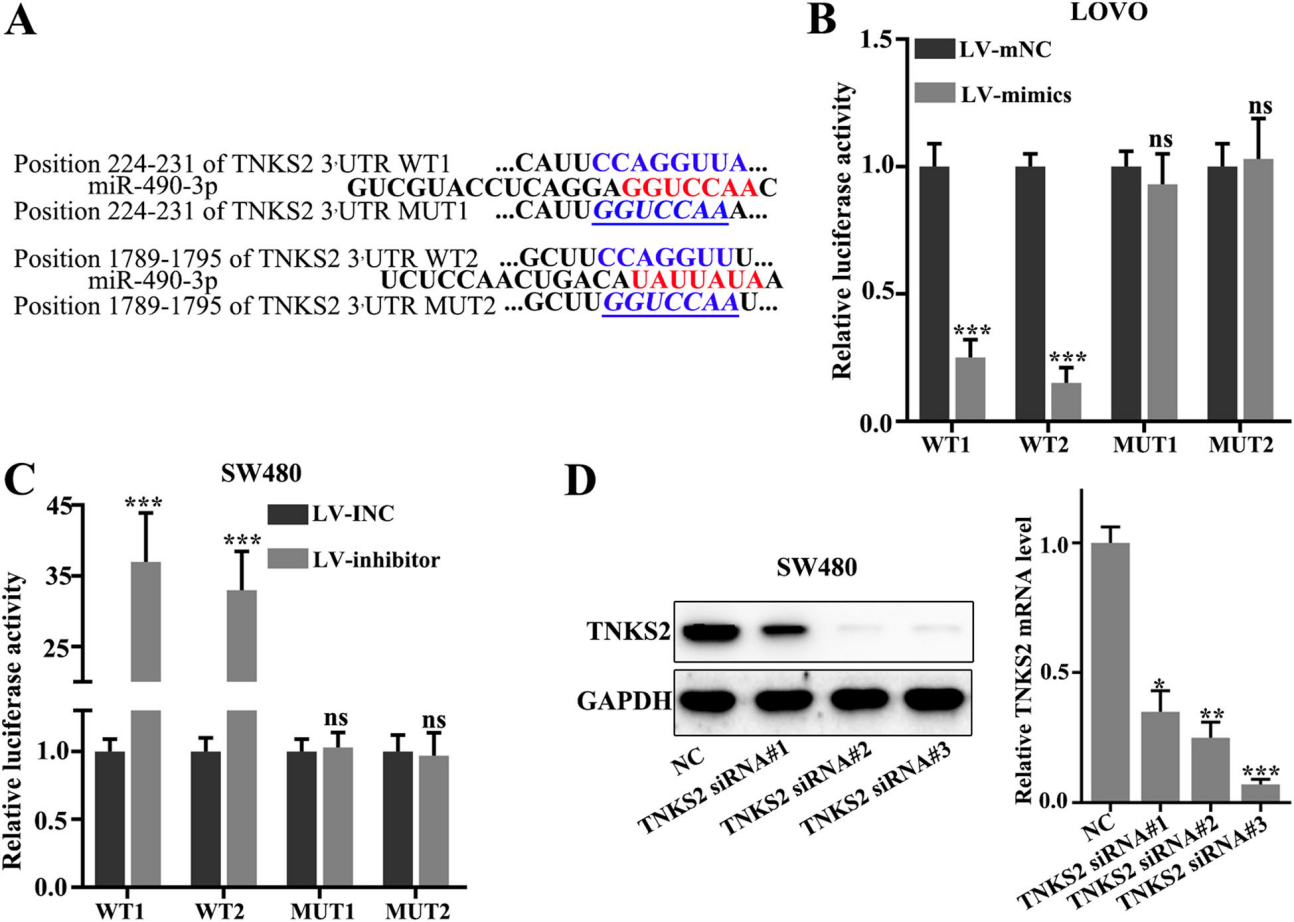
TNKS2 was the downstream target of miR-490-3p. **a** The wild-type and mutant plasmids of the TNKS2 mRNA 3′UTR were constructed according to the potential binding sites of the miR-490-3p and TNKS2 mRNA. **b**, **c** The binding of miR-490-3p and mRNA 3′UTR of wild-type TNKS2 was verified by luciferase assay. **d** TNKS2 siRNA sequences were used to establish the knockdown model of TNKS2

The wild-type and mutant plasmids of the TNKS2 mRNA 3′UTR were constructed according to the potential binding sites of the miR-490-3p and TNKS2 mRNA (Fig. 5a). The binding of miR-490-3p and mRNA 3′UTR of wild-type TNKS2 was verified by luciferase assay (*p*< 0.001) (Fig. 5b, c). Also, the knockdown model of TNKS2 was established through siRNA treatments (Fig. 5d).

### miR-490-3p affected the chemoresistance of CRC cells by targeting TNKS2

**Fig. 6:**
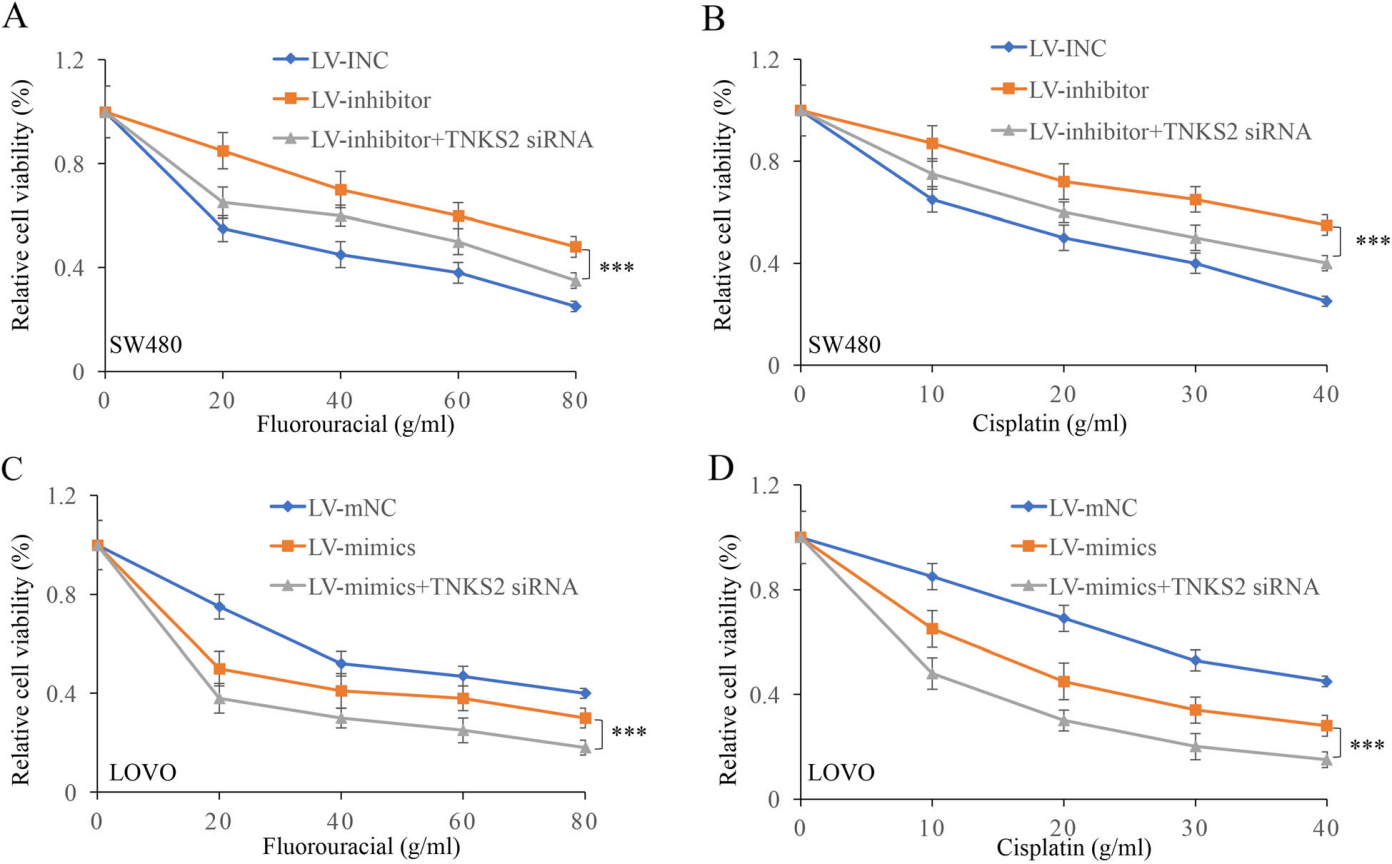
miR-490-3p affected the chemoresistance of colorectal cancer cells through targeting TNKS2. **a**, **b** Group LV-inhibitor+TNKS2 siRNA remarkably suppressed the cell viability of SW480 cells compared with group LV-inhibitor. **c**, **d** Group LV-mimics+TNKS2 siRNA remarkably suppressed the cell viability of LOVO cells compared with group LV-mimics

The chemoresistance of CRC cells was measured after treatment with TNKS2 siRNA and LV-inhibitor or LV-mimics. LV-inhibitor significantly promoted the cell viability of SW480 cell lines compared to that in group LV-INC (*p*<0.001) (Fig. 6a, b). However, simultaneous treatment with TNKS2 siRNA remarkably suppressed the cell viability of SW480 cells. The treatment of TNKS2 siRNA might increase the level of miR-490-3p and inhibit the function of the LV-inhibitor. Besides, simultaneous treatment with TNKS2 siRNA and LV-mimics strengthened the function of LV-mimics and remarkably decreased the cell viability of LOVO (Fig. 6c, d).

## Discussion

miR-490-3p plays a crucial role in regulating tumor occurrence and development in several types of cancers. However, its function in CRC has not been elucidated.

Tankyrases are ubiquitously expressed in human tissues and have tissue-specific sub-locations in cells. For example, in adipocytes, tankyrases locate in the Golgi apparatus. They are overexpressed in several malignancies such as pancreatic cancer. They could regulate the function of proteins involved in miRNA processing [21]. Tankyrases possess multiple ankyrin repeat cluster domains [22]. They can influence a diverse set of cellular activities, such as regulating vesicle trafficking, chromosome activity, telomere structure, and telomerase activity, mediating glucose uptake, and implicating viral replication [23]. Through stabilizing axin, tankyrases have efficacy against the Wnt/β-catenin pathway. Several tankyrase inhibitors have been discovered and became potential drug targets [24].

Tankyrase 2 (TNKS2) can modify substrate proteins by using nicotinamide adenine dinucleotide. TNKS2 locates at 10q23.3 containing five N-terminal ankyrin repeat clusters and can recruit binders and substrates [25]. TNKS2 has multiple intracellular locations (in the cytoplasm and nucleus), such as at the telomeres, centrosomes, and Golgi apparatus [26]. It involves regulating mitotic spindle activity, telomerase function, apoptosis, and Wnt/β signaling pathway, DNA repair, and tumor-suppressive Hippo signaling pathway. Through interfering with telomerase activity, TNKS2 can induce the senescence process of cancer cells [27]. In recent years, TNKS2 has emerged as potentially useful chemical probe for several cancers. For example, Vaiciulis et al. discovered that TNKS2 played crucial roles in laryngeal cancer [28]. Jumatovaite et al. found the participation of TNKS2 in the progression of oral cancer [29]. Nagy et al. revealed that TNKS2 was recruited to DNA lesions [30].

In the current study, TNKS2 was found to be a target gene of miR-490-3p through implementing bioinformatics methods. We examined its expression in 162 CRC specimens and several cancer cell lines. The results revealed that it was downregulated in CRC. Then, we investigated patients with and without metastasis. Results indicated a trend towards the statistical significance of the interrelation between miR-490-3p and metastasis. Data highlighted that CRC metastasis might be attributed to the downregulation of miR-490-3p. It also prevented the migration process of CRC cells. We also evaluated its suppressive role in CRC chemotherapy resistance. It targeted TNKS2 and could bind to TNKS2 mRNA 3′UTR directly. The relative TNKS2 mRNA level was negatively correlated with the expression of miR-490-3p. Taken together, the results of this research brought to light that miR-490-3p inhibited CRC migratory ability as well as chemoresistance through targeting TNKS2.

Fluorouracil and cisplatin are commonly used as anti-tumor drugs. They can inhibit thymine nucleotide synthesis, block the conversion of deoxypyrimidine nucleotide to thymine nucleoside, and interfere with the nucleic acid synthesis in the tumor cells. However, chemoresistance is one of the major problems during treatment with fluorouracil and cisplatin and could further decrease the therapy efficiency. In this study, we demonstrated that overexpression of miR-490-3P and knockdown of TNKS2 could suppress the chemoresistance of LOVO to fluorouracil and cisplatin. Therefore, therapeutic strategies overexpressing miR-490-3P or downregulating TNKS2 might inhibit the chemoresistance of CRC cells.

To sum up, we demonstrated that miR-490-3p expression in CRC cells was low. It can modulate several important pathological processes of CRC cells via targeting TNKS2. This study uncovered novel underlying mechanisms of CRC progression and chemoresistance. The results of our research may attribute to the development of new therapies for CRC.

## Data Availability

The data supporting the study has been presented in the manuscript, and original data could be obtained from the corresponding author through suitable request.
