# Correction: MicroRNA-490-3p inhibits migration and chemoresistance of colorectal cancer cells via targeting TNKS2

**DOI:** 10.1186/s12957-023-03219-y

**Published:** 2023-10-27

**Authors:** Jing Li, Rubing Mo, Linmei Zheng

**Affiliations:** 1 Department of Emergency Surgery, Hainan General Hospital, Hainan Affiliated Hospital of Hainan Medical University, Haikou, 570311 Hainan Province China; 2 Department of Pneumology, Hainan General Hospital, Hainan Affiliated Hospital of Hainan Medical University, Haikou, 570311 Hainan Province China; 3 Department of Obstetrics, Hainan General Hospital, Hainan Affiliated Hospital of Hainan Medical University, Haikou, 570311 Hainan Province China

**Correction: World J Surg Oncol 19, 117 (2021)**

**https://doi.org/10.1186/s12957-021-02226-1**

Following the publication of the original article [1], the author reported that Fig. 2C and E are duplicate. The correct Fig. 2 is included here.

**Fig. 2:**
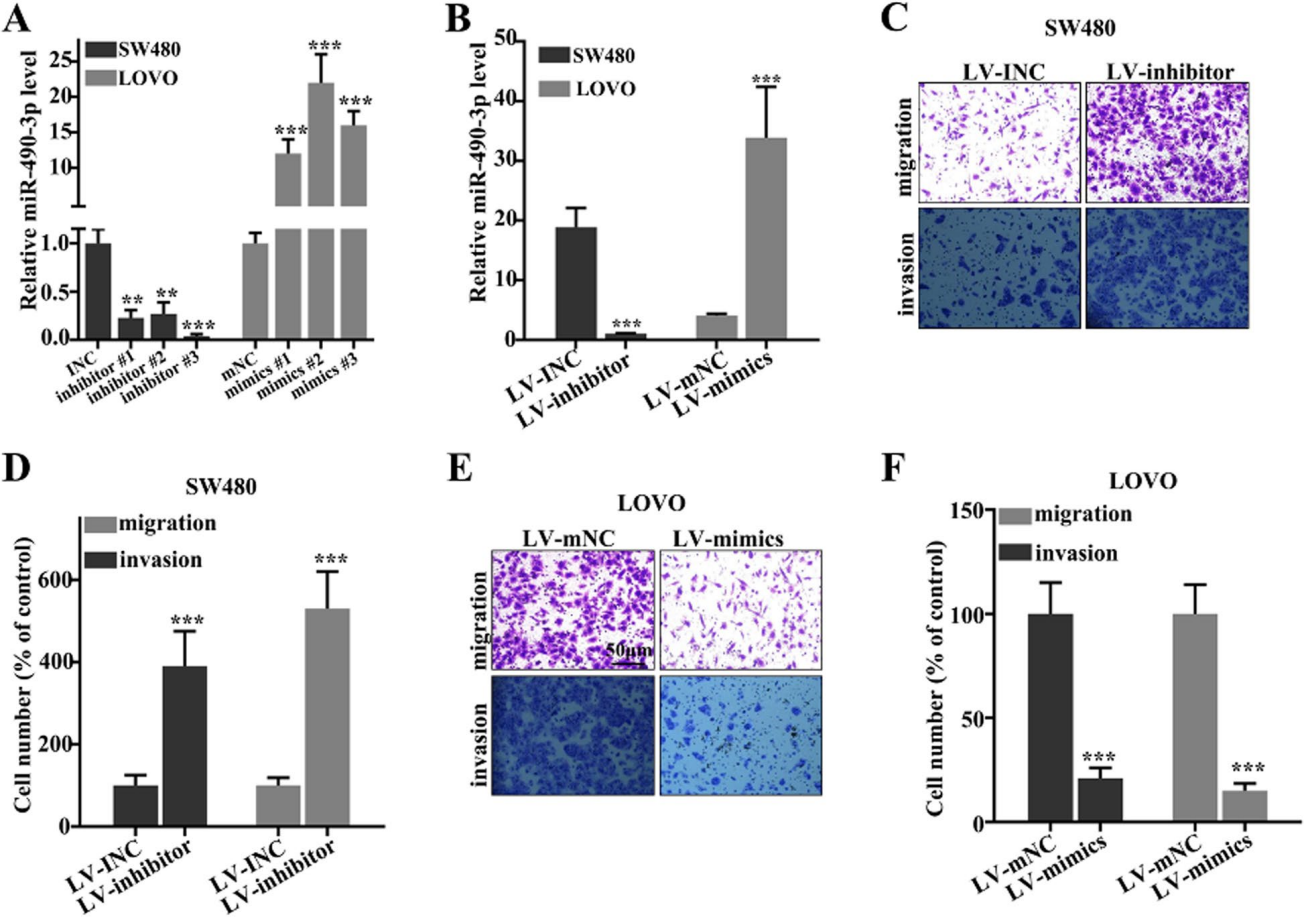
Colorectal cancer cell migration and invasion were suppressed by miR-490-3p. **a** Three mimic sequences and inhibitor sequences were used, and the most effective inhibitor and mimics were selected in the following experiments. **b** The knockdown model of miR-490-3p in SW480 cell line and overexpressed model of miR-490-3p in LOVO cell line were established. **c** The cell migration and invasion of colorectal cancer cells were measured after LV-inhibitor treatment. **d** The cell migration and invasion of colorectal cancer cells were increased significantly after miR-490-3p was knockdown. **e** The cell migration and invasion of colorectal cancer cells were measured after LV-mimics treatment. **f** After treatment with LV-mimics, cell migration and invasion were remarkably suppressed

The original article has been updated.
